# Humoral Response to the HIV-1 Envelope V2 Region in a Thai Early Acute Infection Cohort

**DOI:** 10.3390/cells8040365

**Published:** 2019-04-19

**Authors:** Hung V. Trinh, Neelakshi Gohain, Peter T. Pham, Christopher Hamlin, Hongshuo Song, Eric Sanders-Buell, Meera Bose, Leigh A. Eller, Sodsai Tovanabutra, Nelson L. Michael, Merlin L. Robb, M. Gordon Joyce, Mangala Rao

**Affiliations:** 1 U.S. Military HIV Research Program, Walter Reed Army Institute of Research, Silver Spring, MD 20910, USA; htrinh@hivresearch.org (H.V.T.); ngohain@hivresearch.org (N.G.); ppham@hivresearch.org (P.T.P.); christopher.hamlin@nih.gov (C.H.); hsong@hivresearch.org (H.S.); esandersbuell@hivresearch.org (E.S.-B.); mbose@hivresearch.org (M.B.); leller@hivresearch.org (L.A.E.); Stovanabutra@hivresearch.org (S.T.); nmichael@hivresearch.org (N.L.M.); mrobb@hivresearch.org (M.L.R.); 2 Henry M. Jackson Foundation for the Advancement of Military Medicine, Inc., Bethesda, MD 20817, USA

**Keywords:** HIV-1, V2 region, transmitted/founder (T/F) virus, surface plasmon resonance (SPR), single genome amplification (SGA), RV217 prospective study, CRF01_AE, acute infection, immune response, structural biology

## Abstract

Reduced risk of HIV-1 infection correlated with antibody responses to the envelope variable 1 and 2 regions in the RV144 vaccine trial. To understand the relationship between antibody responses, V2 sequence, and structure, plasma samples (n = 16) from an early acute HIV-1 infection cohort from Thailand infected with CRF01_AE strain were analyzed for binding to V2 peptides by surface plasmon resonance. Five participants with a range of V2 binding responses at week 24 post-infection were further analyzed against a set of four overlapping V2 peptides that were designed based on envelope single-genome amplification. Antibody responses that were relatively consistent over the four segments of the V2 region or a focused response to the C-strand (residues 165–186) of the V2 region were observed. Viral escape in the V2 region resulted in significantly reduced antibody binding. Structural modeling indicated that the C-strand and the sites of viral variation were highly accessible in the open conformation of the HIV-1 Env trimer. V2 residues, 165–186 are preferentially targeted during acute infection. Residues 169–184 were also preferentially targeted by the protective immune response in the RV144 trial, thus emphasizing the importance of these residues for vaccine design.

## 1. Introduction

The RV144 HIV-1 vaccine trial in Thailand demonstrated 31.2% efficacy after 3.5 years [1] and 60% efficacy 1 year after vaccination in post hoc analysis [2]. Immune correlates analysis of a subset of vaccinees at two weeks post final vaccination revealed that antibodies against the Envelope (Env) variable loop 1 and 2 region (V1V2) were associated with lower risk of infection [3]. Further analysis indicated that binding antibody responses to subtypes A, B, C, and CRF01_AE V1V2 proteins also correlated with reduced infection risk [4]. Subsequent sieve analysis of breakthrough infections identified sites of immune pressure at positions 169 and 181 in the V2 region [5]. Epitope mapping of RV144 participant plasma V1V2 antibody responses showed that within V2 vaccine-induced antibodies targeted amino acid residues 169–184 (HXBc2 residue numbering) [6]. 

The Env V1V2-region has extensive loop length, glycosylation variation, and major sequence differences both within and between clades [7,8,9,10,11,12,13,14,15]. The V2 region (amino acids 157–196) is made up of an N-terminal region (157–181) targeted by the majority of the cross-reactive V2 antibodies, while amino acid residues 182–189 form the hypervariable loop at the C-terminal portion. The V1V2-region is located at the membrane-distal apex of the viral Env spike, and in the closed mature HIV-1 molecule [16,17,18] the V1V2-region forms a five-stranded β-sheet motif [19,20,21,22] with significant intra-molecular contacts to the V3-region and inter-molecular contacts to adjacent V1V2-regions. Structures of a set of intermediate forms of the HIV-1 Env trimer have recently been described [23,24,25] which facilitate understanding of the structural plasticity of the V1V2-region. In addition, V1V2-scaffolded molecules in complex with neutralizing antibodies PG9, PG16, PGT145, VRC38.01, BG1, [19,20,21,22,26,27] and poorly or non-neutralizing antibodies 697D, 2297, CH58, CH59, CAP228-3D, and CAP228-16H [15,28,29,30] in complex with V2-peptides have shown the V2 region to exist either in a β-strand or in a helical conformation. The structural definition of the antibody-V2 targeting illustrates the heterogeneity of antibody binding to this region and also the structural plasticity of the HIV-1 V2 region [31]. Two monoclonal antibodies CH58 and CH59 isolated from the RV144 vaccinees recognized linear epitopes in the V2 region (residues 168–182), but with very different structures. CH58 recognized V2 residues 167–176 as a helix and residues 177–181 as an extended coil, while CH59 recognized residues 168–173 as a coil and residues 174–176 as a short 3_10_ helix [29]. These antibodies also induced ADCC [32] in in vitro assays and inhibited the binding of V2 peptide to α4β7 integrin [33]. Antibodies similar to CH58 have been recently isolated from a subtype C infected donor that recognized the same helix-coil V2 conformation as CH58 [15]. Thus, the V2 region appears to be the site of targeted immune response either after vaccination or during HIV-1 infection. Based on the RV144 study, there appears to be a beneficial role for the induction of antibodies to the V2 region of HIV-1 Env. It is possible that the V2 region is also targeted during early acute infection. Insights into the V2-specific antibody responses during acute infection may further reveal sites of vulnerability, providing important information for vaccine design/effective interventions to address the HIV-1 epidemic.

In the present study, we examined a recent prospective natural history study RV217, the “Early Capture HIV Cohort” (ECHO), conducted in Thailand and East Africa [34] to assess the binding responses against homologous and heterologous V2 antigens in the plasma of sixteen Thai participants infected with HIV-1 CRF01_AE during the first year of infection, using surface plasmon resonance (SPR). The natural and limited HIV-1 genetic diversity during the early course of infection in the ECHO study afforded us the opportunity to directly relate induced V2-specific antibody responses to a limited number of genetic variants. We examined transmitted-founder (T/F) HIV *env* sequences from five participants and their relationship to induced V2-specific immune responses during the course of HIV-1 infection. To further deconvolute the V2 antibody responses and their relation to HIV-1 V2 structure, we selected five participants with low or high V2 antibody binding response levels. We divided the V2 region into four designated segments, incorporating viral mutation information based on plasma viral *env* sequence analysis, and assessed the binding to 16-20 mer V2 peptides with 8–10 overlapping amino acid residues. In addition, we assessed the surface-accessibility of the V2 segments in the context of multiple structurally defined conformations of V2 and HIV-1 Env. Overall, these results reveal the specificity of the humoral response to the HIV-1 V2 region in an early infection cohort and the generation of V2-escape mutants, which strongly suggest that the same region is vulnerable during infection and after vaccination. The limited *env* sequence variation during the acute phase of the infection coupled with identification of V2 regions that are targeted early during infection could provide information relevant for vaccine design. A vaccine that targets specific vulnerable areas of the V2 region in the Env might prevent acquisition of the virus and thus generate protective immune responses.

## 2. Materials and Methods

### 2.1. RV217 Plasma Samples

RV217 is a prospective natural-history observational study conducted at four clinical research sites in Uganda, Kenya, Tanzania, and Thailand (1 site/country). The protocol was approved by the local ethics review boards and the Walter Reed Army Institute of Research (RV217, WRAIR 1373, FWA00000373 and IRB00000794) [34]. All individuals participating in this study were at high risk for HIV-1 infection and written informed consent was obtained from all participants. The early capture acute HIV-1 infection (AHI) cohort RV217 has been previously described in detail [34,35]. Briefly, HIV-uninfected participants were evaluated twice weekly with NAT (Aptima HIV-1 RNA Qualitative test, Hologic Inc., San Diego, CA) and analyzed within 24–48 h of sample collection. All confirmed HIV-1 positive participants were referred to care providers for management of the infection, based on national guidelines. Treatment was generally made available at no cost through host nation care and treatment programs.

Participants who tested positive for HIV-1 RNA during the surveillance phase of the protocol and with confirmed HIV-1 infection were enrolled in the long-term follow-up phase. The current analysis focuses on a subset of 5 out of 16 participants from Thailand. None of the participants included here initiated antiretroviral treatment within the timeframe of the current analysis.

### 2.2. Surface Plasmon Resonance Assay (SPR)

Plasma samples from 16 RV217 participants from Bangkok, Thailand, collected pre- and post-infection were analyzed for the presence of binding antibodies by surface plasmon resonance. Plasma samples were heated at 56 °C for 45 min followed by centrifugation at 16,000× *g* at 4 °C for 20 min. The supernatant was stored at −20 °C in small aliquots. Plasma samples were analyzed for the presence of V2-specific antibodies by SPR using a Biacore 4000 system (GE Healthcare, Uppsala Sweden) as previously described with some modifications [36,37,38,39,40]. Standard amine coupling immobilization method and buffer (10 mM Hepes buffer, 150 mM NaCl, and 0.005% Tween-20) was used. Briefly, the CM5 or CM7-S series chip (GE Healthcare, Uppsala, Sweden) surface was activated with a 1:1 mixture of 0.4 M 1-ethyl-3-(3-dimethylaminopropyl) carbodiimide hydrochloride (EDC) and 0.1 M N-hydroxysuccinimide (NHS) for 600 s followed by coupling of 1 µM Streptavidin in 10 mM sodium acetate pH 4.5 for 720 s. The immobilized surface was then deactivated by 1.0 M ethanolamine-HCl pH 8.5 for 600 s. Spot 3 in each flow cell was left unmodified to serve as a blank reference. After the surface deactivation, N-linked biotinylated peptides synthesized by JPT Peptide Technologies GmbH (Berlin, Germany) were injected onto the immobilized streptavidin with various densities. Heat-activated human plasma samples diluted 1:100 in running buffer (10 mM Hepes, 300 mM NaCl and 0.005% Tween 20, pH 7.4) were injected in quadruplicate onto the peptide-captured surface. The complexes bound on the captured surface were then enhanced with a 200 s injection of 30 µg/mL secondary sheep anti-human IgG antibody (Binding Site, Birmingham, United Kingdom). To regenerate the antibody-bound surface, 125 mM HCl was injected for 45–60 s. All injections were conducted at flow rate of 10 µL/min at 25 °C. The data were evaluated using Biacore 4000 Evaluation Software v4.1 (GE Healthcare, Uppsala Sweden). The reported response units (RU) for the IgG-specific values are the difference between the average value of a 5 s window taken 60 s after the end of the anti-IgG injection and the average value of a 5 s window taken 10 s before the beginning of the anti-IgG injection. The response units were double subtracted (unmodified surface, and buffer). The data (RU) are presented as dot plots for individual plasma samples or as RU for the various time points.

### 2.3. Viral Genome Sequencing

Viral RNA was extracted from plasma using the QIAamp Viral RNA Mini Kit (QIAGEN, Valencia, CA, USA). Complementary DNA (cDNA) was synthesized using the SuperScript III RT kit (Invitrogen/Thermo Fisher Scientific, Waltham, MA, USA) following the manufacturer’s instructions. cDNA was amplified as a full genome or 2 half genomes overlapping by 1.5 kb as previously described [41] using single genome amplification (SGA) strategy [42]. Approximately 10 HIV genome sequences were retrieved from each subject at each of the 3 or more visits.

HIV-1 sequences were first aligned with the HXBc2 sequence using HIVAlign via a Hidden Markov Model (https://www.hiv.lanl.gov/content/sequence/VIRALIGN/viralign.html). The aligned sequences were further visualized and refined manually on Geneious Pro 5.6.7, (Newark, NJ, USA) year 2013 (https://support.geneious.com/hc/en-us/articles/227534768). Amino acid sequences of gp160 were extracted into C1, C2, C3, C4, C5, V1, V2, V1V2, V3, V4, V5, gp120, gp140, and membrane-proximal external region (MPER) using python scripts. The genetic distances within subjects were calculated based on the GTR substitution model using Molecular Evolutionary Genetics Analysis (MEGA) 5.0. The V2 region sequence from the five RV217 participants was sub-divided into four segments and peptides of 16 or 20 amino acid residues in length with overlapping 8-10 residues were synthesized. The V2 peptides for each participant was selected based on the CRF01_AE T/F virus and representative CRF01_AE sequences retrieved from each of the 3 or more visits following first positive test for HIV-1 RNA. The sequences have been submitted to GenBank (Accession numbers MK656525-MK656857).

### 2.4. Structural Modeling and Analysis

All figures were generated using PyMol. The V2 region of the HIV-1 Env is shown in two conformations, sheet conformation (PDB ID: 5FYJ) and helical conformation (PDB ID: 6FY1). Structure-based alignment and calculation of the surface area differences (Å^2^) were determined using PyMol. The clade G strain X1193.c1 HIV-1 SOSIP structure (PDB code: 5FYJ) was used to illustrate the HIV-1 glycan shield in the “closed” conformation. The clade B strain B41 HIV-1 SOSIP structure (PDB code: 5VN8) was used to model the HIV-1 Env in the “open” form. Not all glycans were visible in the experimentally determined “open” structure, so Man-5 ((Man)_5_ (GlcNAc)_2_) glycans were modelled at each of the N-linked glycosylation sequons for all three Env protomers using COOT. The atoms of the glycans were modelled with two radii (1.4 and 5.0 Å) to illustrate (i) a single location of the glycans, and (ii) the area covered by the mobile nature of the glycans.

### 2.5. Statistical Analysis

Statistical tests were performed using GraphPad Prism v7 (San Diego, CA, USA) using a spearman non-parametric correlation test assuming a two-tailed distribution.

## 3. Results

### 3.1. V2-Specific Antibodies in Acute HIV-1 Infection

**Figure 1:**
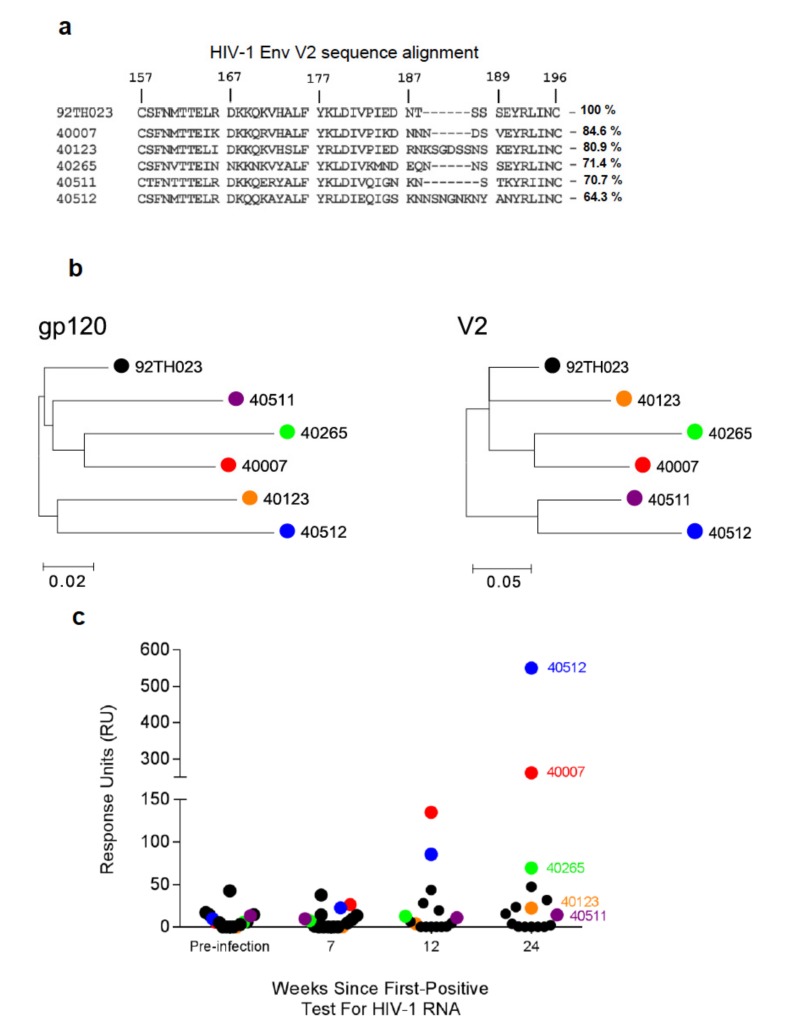
V2-specific antibody responses in the plasma of RV217 Thai cohort participants. (**a**) Sequence alignment of the V2 region for 92TH023 (clade A/E) and CRF01_AE (clade A/E) transmitted founder virus (T/F) sequences derived from five RV217 participants (40007, 40123, 40265, 40511, and 40512) one-week post-infection (HXBc2 numbering is shown for reference). Percentage sequence identity of T/F V2 region amino acid sequence to 92TH023 is indicated to the right of the RV217 V2 sequences. (**b**) Maximum Likelihood (ML) trees showing the phylogenetic relationship among selected RV217 participants. The trees were constructed using the general time reversible model for the participant’s gp120 (left) or V2 (right) sequences (**c**) Plasma binding responses specific to 92TH023 cyclic-V2 peptide were assessed by surface plasmon resonance (SPR-Biacore) for sixteen RV217 participants, prior to infection and at 7, 12, and 24 weeks following first positive test for HIV-1 RNA. Each circle represents the mean response units (RU) of four independent measurements of plasma samples diluted 1:100 for each of the 16 participants. Colored spheres indicate the five participants selected for further analyses.

To determine if the V2 region was a target for the induction of antibodies during a primary infection, plasma samples from an early capture AHI prospective study (RV217) were examined. Plasma samples (1:100 dilution) from 16 participants from the study located in Thailand, who became infected with CRF01_AE were analyzed (pre-infection and at 7, 12, and 24 weeks following first positive test for HIV-1 RNA) by SPR for the presence of V2-specific antibodies using N-linked biotinylated full-length cyclic V2 peptide based on the sequence of CRF01_AE strain 92TH023 (Figure 1a). Maximum Likelihood (ML) trees showing the phylogenetic relationship was constructed using the general time reversible model for the participant T/F gp120 or V2 sequences (Figure 1b). Samples from weeks 7 and 12 were chosen to represent the V2 antibody responses at the early time points following infection. V2-specific antibody responses were much higher at week 24 compared to week 12 post-infection (Figure 1c) time point and the availability of ~ week 24 *env* sequencing data showed variations in the sequences. The V2 sequences from each of the 5 subjects one-week post-infection showed 64–85% identity with the 92TH023 peptide sequence used to measure V2 binding antibody responses (Figure 1a). Seven of the 16 participants did not exhibit V2-specific IgG antibodies, whereas varying degrees of binding were observed in the 9 other participants (Figure 1c). The three participants (40512, 40007, and 40265) with the highest V2 antibody binding responses, and two participants (40123 and 40511) who showed detectable but low V2 binding responses at week 24 were selected (Figure 1c) for further analysis.

### 3.2. Single Genome Analysis of Longitudinal HIV-1 Env Sequences

**Figure 2:**
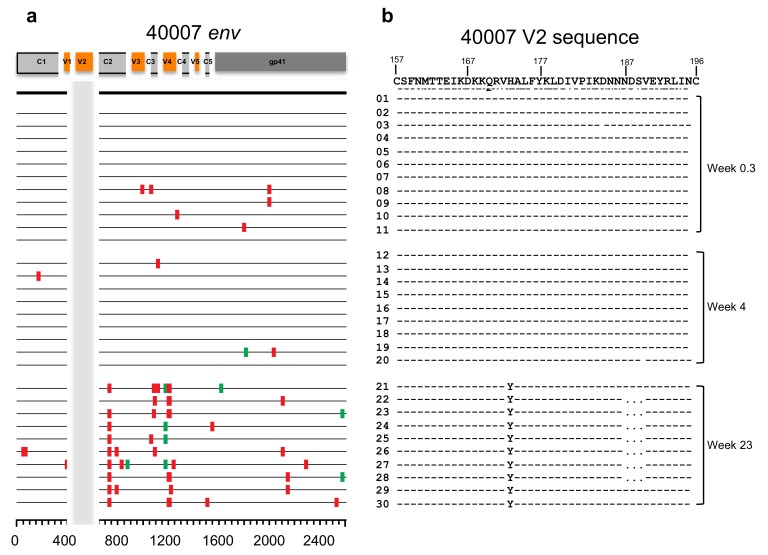
HIV-1 *env* and V2 sequences of RV217 participant 40007 at weeks 0.3, 4, and 23 post-infection. (**a**) Highlighter plot of HIV-1 *env* sequences from participant 40007 with changes from the consensus sequence (black line on the top represents the inferred T/F sequence at week 0) demarcated by red (synonymous) or green (non-synonymous) vertical markers. The V2 region is indicated by the vertical orange bar. (**b**) Sequence alignment of the HIV-1 Env V2 protein sequence from ~10 single-genome amplification (SGA) sequences for each time point. Sequence variation are indicated by the newer variant amino acid letter, and deletions are indicated by dots. The numbers on the master sequence show the HXB2 location.

To further investigate the association of viral sequence evolution and development of V2-specific binding antibodies, ten single genome amplification and sequencing (SGA) analysis and sequence alignment for the V2 region of T/F viruses were conducted at each of three or four time points (weeks 0–1, 4, 23–25, and 52 post-infection). Sequence reads were aligned for the five selected participants, 40007 (Figure 2), 40123 (Figure S1), 40511(Figure S2), 40265 (Figure S3) and 40512 (Figure S4). In the case of participant 40007 (Figure 2), there was no change in the viral sequence within the V2 region during weeks 0.3 and 4 post-infection, in contrast to other regions of the envelope. At week 23 post-infection, two V2-region variants were observed. There was a fixed single amino acid change His173Tyr in all 10 sequences analyzed (100% frequency). This substitution was the only one fixed in Env at that time, indicating a strong positive selection targeting this position (Figure 2). In addition, 7 out of the 10 sequence reads (70% frequency) also showed a three-amino acid (Asp-Ser-Val) deletion at positions 188–190 (Figure 2b). Participant 40123, who was infected with two distinct T/F viruses showed viral variation in multiple regions of the Env starting as early as week 0.9 and continuing into weeks 4 and 21 (Figure S1). The two T/F lineages had 6 amino acid differences in V2, and the minor lineage was detected in only 1 out of 14 sequences at week 0.9. At week 4, all of the V2 sequences were identical to the major T/F sequence (Figure S1). A single amino acid change from the major T/F virus (Arg189Ser), likely derived from the minor T/F virus was observed in 90% of the V2 sequences analyzed at week 21 post-infection (the “Ser” is more likely derived from the minor T/F through recombination, than a de novo substitution based on the *env* sequences at week 21 that appear to be very closely related to the minor T/F) (Figure S1b). Participant 40511 (Figure S2) showed a single amino acid variation Asn185Asp at week 23 in all 10 sequences analyzed (Figure S2b). In contrast, participant 40265 (Figure S3) did not show any dominant amino acid variants within the V2 region, with only single amino acid changes in single SGA sequences for each of the weeks 3.9 and 23 post-infection (Figure S3b). Participant 40512 (Figure S4), showed several mutations and deletions at week 22. At week 58, a super-infecting strain was first detected that dominated the viral population. The superinfecting strain sequences were consistently seen in 76% of the V2 sequences, while 24% of the V2 sequences represented the original virus (Figure S4b). In all participants, significant changes in viral sequences in the V2 region were consistently seen around the 6-month time point.

### 3.3. Design of HIV-1 V2 Peptides Based on Viral SGA Sequences

**Figure 3:**
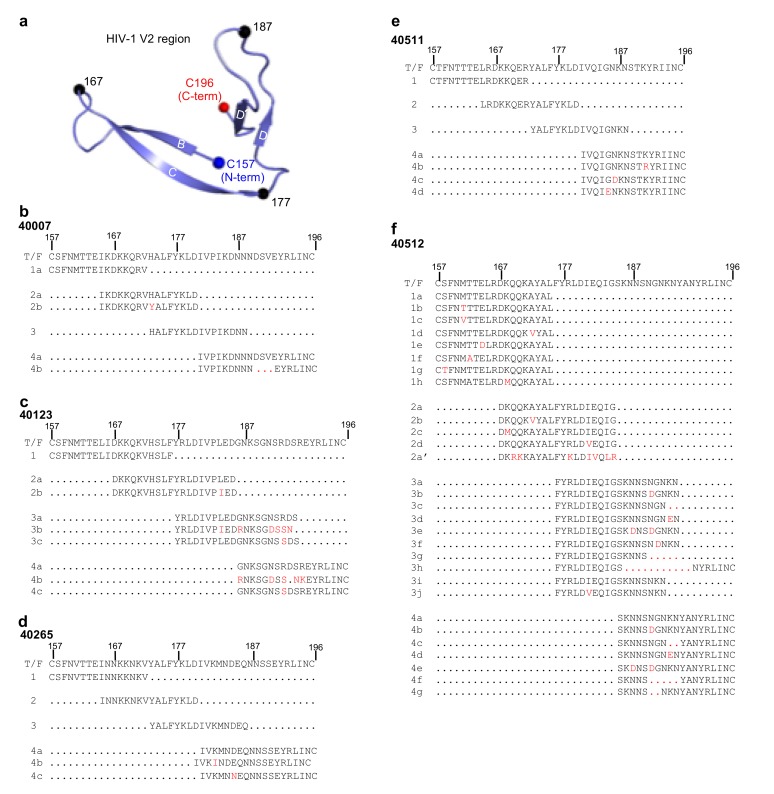
Design of HIV-1 Envelope V2 peptide segments for five RV217 participants. (**a**) Ribbon representation of the HIV-1 V2 region (residues 157–196) from the X1193.c1 SOSIP.664 Env trimer structure (PDB ID: 5FYJ). The N-termini (blue), C-termini (red), and every tenth residue (black) are represented as spheres. (**b**–**f**) Sequence alignment of the V2 region for five RV217 participants (40007, 40123, 40265, 40511, and 40512). The V2 region from each participant was sub-divided into four segments (1–4) and peptides, with overlapping 8-10 residues were synthesized. HIV-1 V2 peptides were selected based on the T/F and representative viral sequences determined at approximately weeks 1, 4, and 24 following first positive test for HIV-1 RNA, incorporating viral mutations (red) and deletions (red dots) at these time points. In participant 40512, peptide 2a’, represents the V2 segment 2 sequence following a second CRF01_AE virus infection that occurred in this participant between weeks 47 and 57.

A ribbon representation of the HIV-1 V2 region (residues 157–196) based on the fully glycosylated HIV-1 Clade G X1193.c1 SOSIP.664 Env trimer structure (PDB ID: 5FYJ) is shown in Figure 3a. To assess whether a particular region of the V2 was preferentially targeted by the immune system to generate antibodies and if this in turn induced a selection pressure on the virus to generate escape mutants, a set of V2 peptides were generated. Based on the T/F virus V2 region sequence and the SGA sequences from each of the five selected participants, overlapping peptides (each 16–20 amino acids in length with 8–10 overlapping amino acid residues) incorporating SGA-identified mutations (red color letter), or deletions (red dots) were synthesized, (Figure 3). The V2 region was divided into 4 segments (1–4) and each segment had variants (1a, 2a, 2b, etc.) that included the sequence of the T/F and any SGA-identified variants in that segment (Figure 3b–f). Peptides for participant 40007 were generated based on the T/F viral sequence (1a); segment 2, containing T/F viral sequence (2a) and a mutant His173Tyr (2b) which was found at week 23 post-infection (Figure 2); segment 3; and segment 4a, comprising T/F viral sequence, and 4b, comprising the Asp-Ser-Val deletion that was observed at 23 weeks post-infection (Figure 2 and Figure 3b). This naming system was also used for the design of peptides for the other four participants (Figure 3c–f). Any Env V2 variants detected in the SGA sequences were included in the peptide sequences, even if they were only observed at a single time point. In contrast to the other four participants, participant 40512 (Figure 3f and Figure S4) had a higher rate of variants in the V2 region, which were first observed sporadically at 3.7 weeks post-infection, but more consistently at week 22. A series of overlapping peptides were designed for each of the 4 segments, segment 1 (1a–h), segment 2 (2a–d), segment 3 (3a–j), and segment 4 (4a–g). In addition, this participant was infected with a second CRF01_AE strain (super-infection) and an additional segment 2 peptide (2a’) based on the SGA sequencing at week 58 attributed to the super-infection was also assessed.

### 3.4. Binding Responses to V2 Region Peptides

**Figure 4:**
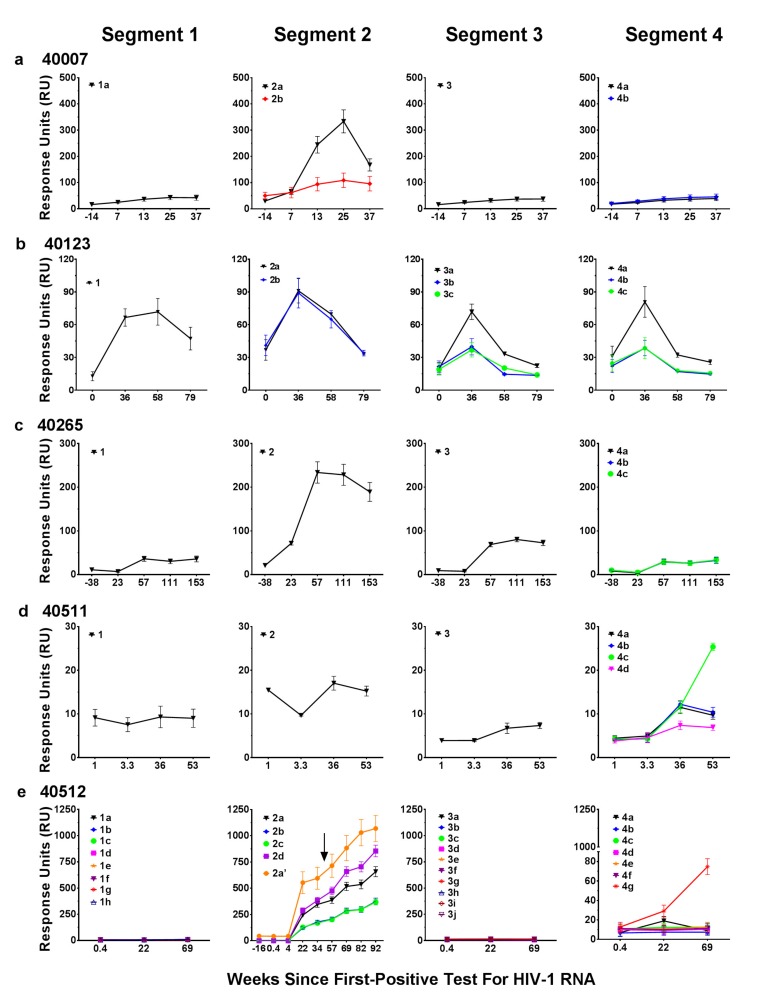
Autologous antibody binding responses to the four V2 segments in RV217 participants. (**a**–**e**) Plasma antibody binding responses to V2 peptides for the four V2 segments (shown in Figure 3) were measured by SPR-Biacore from prior to infection and following HIV-1 infection (first positive test for HIV RNA). In the case of participant 40512, super-infection with a second CRF01_AE virus was detected between weeks 47 and 57 (denoted by an arrow). Peptide 2a’ is representative of the V2 segment 2 sequence of the super-infecting virus. Segments 2 and 4 in Figure 4e, 2b (blue) and 4a,b (black and blue), respectively, are hidden by overlapping data. The data shown are the mean response units ± S.D of four independent measurements for each of the five participants at each time point. The response measurement for each peptide is designated in different colors.

The various biotinylated linear peptides for the five participants were synthesized and used in a SPR-Biacore assay to measure the antibody binding profile of the plasma samples with the aim of determining the influence of V2 sequence changes on antibody responses, identification of antibody escape, and mapping the targeted response within the V2 region (Figure 4). All of the participants showed antibody targeting to the V2 peptide segments. Minimal to no antibodies were present in the plasma of all participants to V2 peptide segment 1, except for participant 40123. Plasma samples from four out of the five participants exhibited reasonable binding antibody responses to segment 2. In participant 40007, the antibody response was predominantly directed to segment 2, with minimal binding to segments 1, 3, and 4 (Figure 4a). The antibody response to segment 2 (peptide 2a) showed a peak at week 25 followed by a decline in antibody responses by week 35 (Figure 4a). The decline in antibody responses coincided with a consistent single amino acid mutation His173Tyr (peptide 2b) in the virus at week 23 post-infection (Figure 2) compared to the T/F virus (peptide 2a). In addition, there was a deletion of 3 amino acids Asp-Ser-Val in segment 4 in this viral variant. The single amino acid change in segment 2 in participant 40007, resulted in a dramatic 4-fold decrease in the magnitude of the antibody response (Figure 4a). However, deletion of amino acids Asp-Ser-Val (peptide 4b) did not affect the antibody response when compared to the T/F virus segment 4 (peptide 4a), while minimal antibody responses were observed to segment 4.

In participant 40123, a V2 segment 2 variant Leu184Ile (peptide 2b) did not affect the antibody response compared to peptide 2a (Figure 4b). However, antibody responses to peptide 3b, which included all of the SGA-observed variants in segment 3, or peptide 3c, which included only 1 of the segment 3 mutations (Arg189eSer) resulted in approximately a 3-fold lower antibody response compared to the corresponding T/F 3a peptide (Figure 4b). Peptides 4b and 4c (segment 4) also resulted in approximately a 3-fold lower antibody response compared to the corresponding T/F 4a peptide (Figure 4b). Based on the peptide sequences it appears that a single amino acid Arg at position 189e is a critical amino acid in segment 4 as a change to Ser represented in peptides 3c and 4c resulted in significant decreases in the peak antibody response. This single amino acid change to Ser was consistently seen in 90% of the SGA sequence reads (Figure S1b).

In the case of participant 40265, the antibodies were directed mainly to segment 2 of the V2 region (Figure 4c). Single amino acid changes in peptides 4b and 4c (Figure 3d), which were variable and low frequency in the SGA sequencing (Figure S3b), did not alter the antibody response compared to the corresponding T/F peptide 4a (Figure 3d). Although the responses were low or at background levels in participant 40511 to all segments of the V2 region, a single amino acid mutation from Asn185Asp (peptide 4c) resulted in approximately a 2.5-fold increase in the antibody response (Figure 4d).

In participant 40512, there was no binding response to segments 1 and 3, nor to the majority of segment 4 peptides despite multiple variants tested in these segments (Figure 4e). Segment 2 induced the highest and most variable antibody response. The super-infecting variant was first detected at week 57 (visit 17, day 401). It was not detected at the previous visit (visit 16, week 47, day 330) and at earlier time points. Additional binding data from later time points were included since participant 40512 showed super-infection (this was not observed in the other participants) and includes binding to peptides based on *env* sequences prior to and following super-infection. Peptide 2a’, derived from the super-infection V2 segment 2 sequence, showed robust binding responses from time of initial infection indicating that the V2 response in participant 40512 had heterologous specificity early in infection (Figure 4e).

**Figure 5:**
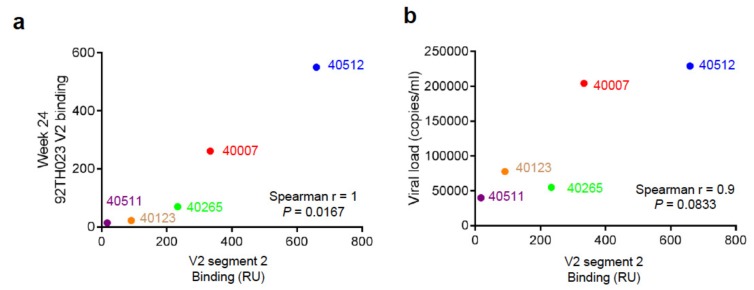
Statistical analysis of V2 binding, V2 segment 2 binding, and viral load titer. (**a**) Correlation of 92TH023 V2 plasma response with peak RV217 T/F V2 segment 2 binding response. (**b**) Correlation of RV217 participant viral load with RV217 T/F V2 segment 2 binding response at week 24. Spearman non-parametric correlation was performed assuming a two-tailed P value.

The results from the five participants indicate a range of binding responses targeting the V2 region segment 2 derived from the T/F and later variants and demonstrate how changes in the viral V2 sequence influences the antibody response. Analysis of the V2 segment 2 binding response and the 92TH023 V2 binding response at week 24 post-infection indicated a significant correlation as determined by Spearman non-parametric analysis (Figure 5a). V2 binding responses to 92TH023 at week 24 post-infection for the 5 participants plotted against the viral load measured closest to that time point showed no significant relationship between V2 responses and viral load. Statistical analysis using a Spearman correlation two tailed test resulted in r = 0.9, and *P* = 0.0833, which indicated a trend towards positive correlation (Figure 5b). We speculate that the apparent correlation may indicate that high viral load leads to increased responses to the V2 region, based on the total antigen amount; i.e., higher levels of antigen induced higher immune responses as opposed to V2 response leading to higher viral load.

### 3.5. Structure Modeling of the HIV-1 V2 Region

**Figure 6:**
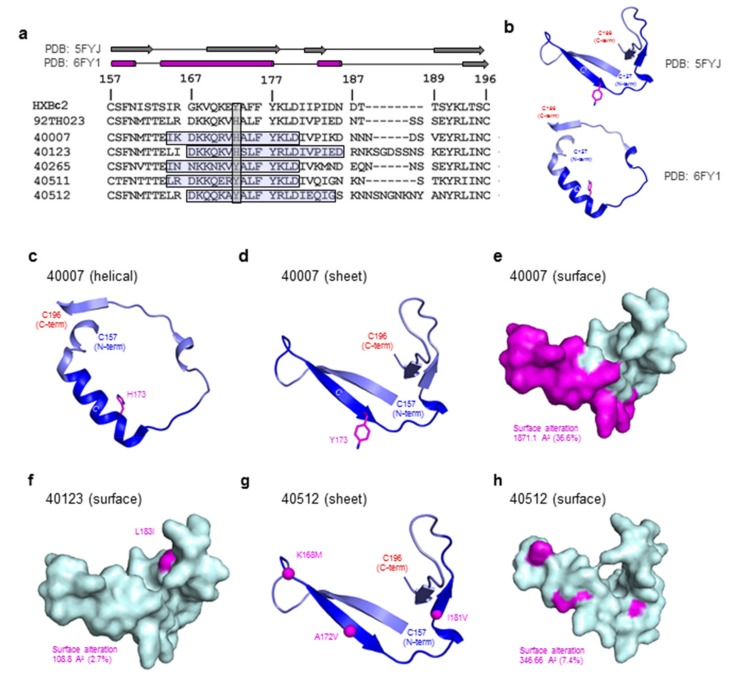
Modeling of the HIV-1 Env V2 region. (**a**) Sequence alignment of RV217 participant T/F sequences with HXBc2, and 92TH023. A schematic of the secondary structure of two representative structures of the V2 region are shown above the sequence. Residue 173 is highlighted in the sequence alignment, and the sequence defined as the V2 segment 2 for each participant is demarcated by a horizontal box. (**b**) Crystal structures of the HIV-1 Env V2 region shown in ribbon representation. (**c**,**d**) Participant 40007 HIV-1 Env V2 region is modeled with strand C (residues 168–174) in a helical (**c**) or strand (d) conformation. Segment 2 is colored dark blue. (**e**,**f**,**h**) surface representation of the T/F V2 region from participants 4007, 40123, and 40512, respectively. In the case of 40007, the V2 region that differs between the helical and strand forms is highlighted in magenta. (**g**) Participant 40512 V2 region is modeled and shown in cartoon representation, with residues within segment 2 that vary over the course of the study indicated in magenta. Surface area changes are indicated in Å^2^. The N- and C-termini of the V2 region are marked in blue and red, respectively.

To understand the impact of the viral variation on the V2 region structure, we carried out structure modeling of the V2 region for three participants, 40007, 40123, and 40512 based on the sequence data available at the 6-month (~week 25) time point (Figure 6). Based on the sequences available in the LANL database, Tyr173 is present in the majority of subtype B (72%) and subtype C (68%) sequences, whereas in CRF01_AE, the frequency of histidine is slightly higher than tyrosine at this position (46% vs. 44%). In the T/F virus from RV217 participants 40007 and 40123, residue 173 is a histidine (Figure 6a). Structural characterization of 92TH023 V2 peptide bound to monoclonal antibody CH58 isolated from a RV144 vaccinee demonstrated that the V2 peptide can form a helical conformation [29]. In addition, the recent structure of CAP16H in complex with a V1V2-scaffold allowed visualization of an extended helical form of the V2 region (Figure 6b). Based on the helical (PDB ID: 6FY1) and sheet (PDB ID: 5FYJ) structural variants we modeled the participant V2 region variants in both conformations (Figure 6c–h). A shift in the overall structure from sheet to helical form would result in significant alteration of the surface (36.6%) of the V2 region (Figure 6d,e). In the case of participant 40007, at week 23, a His173Tyr dominant variant was observed, and based on the dramatic change in binding seen with this variation, we hypothesize that the His173Tyr results in a shift in the overall conformation of the V2 region. Concomitant with this mutation was a decrease in the magnitude of the antibody response and the generation of escape variants. In participant 40123, a Leu183Ile variant (2.7% surface variation) is modelled (Figure 6f), while participant 40512 Env variants Lys168Met, Ala172Val, and Ile181Val result in a surface alteration of 7.4% (Figure 6g,h). These surface alterations match the resulting small changes in the associated participant plasma binding levels. In the other two participants, 40265, and 40511, the V2 region showed minimal sequence variation, which also matches to the consistent V2 region plasma binding results.

**Figure 7:**
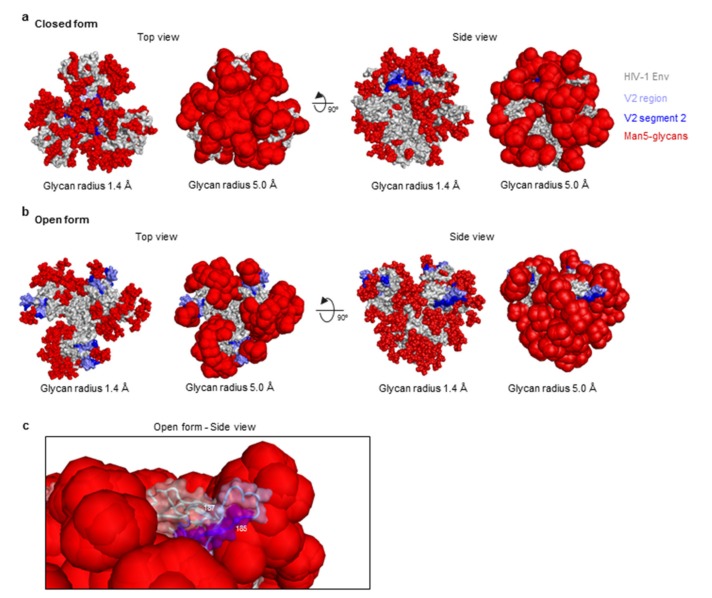
Modeling of HIV-1 Env V2 segment 2 surface accessibility. (**a**) Surface representation of the “closed” form of the HIV-1 Env trimer (PDB ID: 5FYJ) in two orientations with top and side views. The V2 region (residues 157–196) is colored light blue, and segment 2 in dark blue. HIV-1 Env Man5-glycans are modeled with two radii (1. 4 Å, and 5.0 Å) and colored red. (**b**) HIV-1 Env in the “open” form (PDB ID: 5VN8) is shown in surface representation with glycans and V2 depicted as in (**a**). (**c**) Close-up view of the V2 region (blue) in the “open” conformation in two views.

To determine the accessibility of the V2 region within the trimer in both closed (Figure 7a) (PDB ID: 5FYJ) and open (Figure 7b) forms (PDB ID: 5VN8), we modeled the glycosylated HIV-1 Env trimer with the glycan shield displayed at two different radii 1.4 Å (actual atomic radii) and 5.0 Å (a surrogate for the mobile nature of the HIV-1 glycan shield). In the native pre-fusion form (Figure 7a), the V2 region (blue color) is largely occluded by the glycan shield (red color) and is barely visible, indicating that it would be minimally accessible to the humoral response and thus weakly immunogenic. In contrast, the open form of the HIV-1 Env (Figure 7b), allows significant accessibility to the V2 region, and in particular the V2 region segment 2 is surface accessible, indicating antigenic availability. 

## 4. Discussion

Prospective HIV-1 infection cohorts, such as RV217, provide an opportunity to assess humoral response in the setting of limited viral diversity during the early stages of HIV-1 infection. It also allows examination of how viral variants can escape the early antibody immune pressure. The continuous and early sampling schedule in the RV217 study [34] allowed us to determine humoral binding responses, combined with viral sequence information at the time of initial infection, and at multiple time points throughout the first year. Despite similar levels of HIV-1 viral setpoint and HIV-1 strain sequence diversity in Env and the V2 region, antibody binding responses to a heterologous V2 sequence showed significant variation from non-reactive to highly reactive. This variation in reactivity is similar to that seen in the RV144 vaccine trial, where the immunization regimen elicited significant variation in V2 immune responses. Single genome analysis of the HIV-1 virus from time of infection during the first year showed a low level of viral sequence variation throughout the *env* sequence in the first four weeks of infection. However, by the six-month time point, there existed variable but significant sequence diversity resulting in changes in the V2 region amino acid sequence. In participant 40265, targeted deep sequencing analysis detected a minor variant first at day 7 post-infection which remained detectable at low levels (1.1–4.5%) through 3 months post-infection. However, the minor variant contributed only marginally (1.1%) to changes in the Env region even after 6 months post-infection [41]. It is likely that cytotoxic T lymphocyte targeting observed during the first month of infection was the driving force of escape variants [41], as opposed to early immune pressure from V2 antibodies; hence, there was minimal sequence variability in the V2 region. In the case of participant 40512, where an HIV-1 super-infection occurred between weeks 47 and 57, there was dramatic sequence variation throughout *env* and the V2 region following the super-infection event.

Given the variation in V2 immune responses, we wanted to understand in detail and to de-convolute the V2 binding response. We divided the V2 region into four segments, and measured binding to a set of peptides with T/F sequence and other variants identified by the SGA sequencing. In general, there were two patterns of immune targeting, (i) a uniform response across the four segments of the V2 region, and (ii) a response targeted to the strand C segment (residues 165–186) of the V2 region. The peak of the immune response to the strand C segment varied between the participants, and while this study only analyzed five individuals, and a limited number of antigens based on the SGA sampling performed only from the plasma compartment, intriguingly, there was a significant correlation between the response to the strand C segment and response to the heterologous 92TH023 V2 region, indicating a relationship between heterologous V2 binding and autologous V2 segment 2 binding. There is no obvious sequence determinant that explains this relationship. In general, the immune response to the strand C region was not dramatically affected by sequence variation, with the exception of participant 40007, where a single V2 mutation caused escape variants that exhibited significantly reduced binding of the peptide. It is intriguing that mutations in the V2 region were observed only around the 6-month time point, despite mutations in other regions of the envelope that were observed earlier in infection. We hypothesize that the increase in V2-specific antibodies by week 24 post-infection probably caused the virus to mutate to escape the immune pressure from the antibodies. It was not possible to follow the responses at later time points as this participant began ART treatment.

The V2 region is a significant target for the immune response and known determinant of neutralizing and non-neutralizing protective responses. Given recent structural information, we assessed V2 structural forms and viral sequence variation, in conjunction with our binding data to try to understand the binding responses. HIV-1 Env has significant plasticity, and structural characterization of the V2 region has shown that this region can exist either as a β-strand or a helical form and is targeted by antibodies in either conformation. There has been much supposition over the transition dynamics of the V2 region from β-strand to helical forms with residue 173 hypothesized to play a significant role in this transition. The recent structure of a V2 scaffold with antibody CAP228-16H indicates that the V2 region can form β-strand or helix structures despite similar or identical sequences [15]. Computational structure-based prediction of the V2 region alongside nuclear magnetic resonance measurements indicates the V2 region has a propensity for a helical form [31,43], while HIV-1 Env structures in the mature closed conformation have all contained the β-strand form of the V2 structure. In addition to the effects of HIV-1 Env sequence variation, this molecular transition would result in dramatic changes in the Env structure significantly muting the immune response to this region. In the case of participant 40007, a single amino acid change His173Tyr results in almost complete loss of antibody binding. Given the single sequence change, we believe that the dramatic reduction in binding is due to structural transition of the V2 region. In the case of participant 40512, significant sequence variation occurs during the first year of infection. Despite this sequence variation, the binding response changes are less than that seen in participant 40007. Within the context of both the “open” and “closed” forms of the HIV-1 Env trimer, the glycan coverage results in significant shielding from the immune response. Strikingly, in the context of the “open” form of Env, the V2 region is surface exposed, and of significance, residues 167–187 are not shielded by glycans even when the mobile nature of the glycans are modelled. This may explain the significant targeting of the V2 region early in infection and may also indicate that vaccine responses can target the V2 region on the virus prior to cell entry.

## 5. Conclusions

The observations presented here indicate that residues 165–186 in the HIV-1 gp120 Env V2 are a key region targeted during acute infection, resulting in heterologous V2 recognition, and match the responses seen in the RV144 vaccine trial. Sieve mutations in the V2 region in the RV144 vaccine trial resulted in escape mutants. Viral mutation and variation in this region resulted in reduced humoral response in this acute HIV-1 infection study cohort. This is clearly seen in at least one participant, 40007 where a single amino acid mutation resulted in a significant conformational change and a loss of antibody binding. Despite this portion of the V2 region (residues 165–186) being consistently targeted, it also has significant structural plasticity, which may lead to immune evasion. Structural modeling indicated that this region is significantly occluded in the closed native, prefusion trimer, while accessible in the CD4-liganded open Env conformation. Since the V2 region is targeted in protective vaccine-elicited responses and early in natural HIV-1 infection where there is limited genetic variability, this region should be carefully considered for future vaccine design and subsequent immune responses.

## Supplementary Materials

The supplementary material is available online at https://www.mdpi.com/2073-4409/8/4/365/s1.

## References

[1] Rerks-Ngarm S., Pitisuttithum P., Nitayaphan S., Kaewkungwal J., Chiu J., Paris R., Premsri N., Namwat C., de Souza M., Adams E. (2009). Vaccination with ALVAC and AIDSVAX to prevent HIV-1 infection in Thailand. N. Engl. J. Med..

[2] Robb M.L., Rerks-Ngarm S., Nitayaphan S., Pitisuttithum P., Kaewkungwal J., Kunasol P., Khamboonruang C., Thongcharoen P., Morgan P., Benenson M. (2012). Risk behaviour and time as covariates for efficacy of the HIV vaccine regimen ALVAC-HIV (vCP1521) and AIDSVAX B/E:a post-hoc analysis of the Thai phase 3 efficacy trial RV 144. Lancet Infect. Dis..

[3] Haynes B.F., Gilbert P.B., McElrath M.J., Zolla-Pazner S., Tomaras G.D., Alam S.M., Evans D.T., Montefiori D.C., Karnasuta C., Sutthent R. (2012). Immune-correlates analysis of an HIV-1 vaccine efficacy trial. N. Engl. J. Med..

[4] Pazner S., deCamp A., Gilbert P.B., Williams C., Yates N.L., Williams W.T., Howington R., Fong Y., Morris D.E., Soderberg K.A. (2014). Vaccine-induced IgG antibodies to V1V2 regions of multiple HIV-1 subtypes correlate with decreased risk of HIV-1 infection. PLOS ONE.

[5] Rolland M., Edlefsen P.T., Larsen B.B., Tovanabutra S., Sanders-Buell E., Hertz T., deCamp A.C., Carrico C., Menis S., Magaret C.A. (2012). Increased HIV-1 vaccine efficacy against viruses with genetic signatures in Env V2. Nature.

[6] Karasavvas N., Billings E., Rao M., Williams C., Zolla-Pazner S., Bailer R.T., Koup R.A., Madnote S., Arworn D., Shen X. (2012). The Thai Phase III HIV Type 1 Vaccine trial (RV144) regimen induces antibodies that target conserved regions within the V2 loop of gp120. AIDS Res. Hum. Retroviruses.

[7] Derdeyn C.A., Decker J.M., Bibollet-Ruche F., Mokili J.L., Muldoon M., Denham S.A., Heil M.L., Kasolo F., Musonda R., Hahn B.H. (2004). Envelope-constrained neutralization-sensitive HIV-1 after heterosexual transmission. Science.

[8] Chohan B., Lang D., Sagar M., Korber B., Lavreys L., Richardson B., Overbaugh J. (2005). Selection for human immunodeficiency virus type 1 envelope glycosylation variants with shorter V1-V2 loop sequences occurs during transmission of certain genetic subtypes and may impact viral RNA levels. J. Virol..

[9] Sagar M., Wu X., Lee S., Overbaugh J. (2006). Human immunodeficiency virus type 1 V1-V2 envelope loop sequences expand and add glycosylation sites over the course of infection, and these modifications affect antibody neutralization sensitivity. J. Virol..

[10] Zolla-Pazner S., Cardozo T. (2010). Structure-function relationships of HIV-1 envelope sequence-variable regions refocus vaccine design. Nat. Rev. Immunol..

[11] O’Rourke S.M., Sutthent R., Phung P., Mesa K.A., Frigon N.L., To B., Horthongkham N., Limoli K., Wrin T., Berman P.W. (2015). Glycans flanking the hypervariable connecting peptide between the A and B strands of the V1/V2 domain of HIV-1 gp120 confer resistance to antibodies that neutralize CRF01_AE viruses. PLoS ONE.

[12] Smith S.A., Burton S.L., Kilembe W., Lakhi S., Karita E., Price M., Allen S., Hunter E., Derdeyn C.A. (2016). Diversification in the HIV-1 Envelope Hyper-variable Domains V2, V4, and V5 and Higher Probability of Transmitted/Founder Envelope Glycosylation Favor the Development of Heterologous Neutralization Breadth. PLoS Pathog..

[13] Ashokkumar M., Nesakumar M., Cheedarla N., Vidyavijayan K.K., Babu H., Tripathy S.P., Hanna L.E. (2017). Molecular Characteristics of the Envelope of Vertically Transmitted HIV-1 Strains from Infants with HIV Infection. AIDS Res. Hum. Retrovir..

[14] Li F., Ma L., Feng Y., Hu J., Ni N., Ruan Y., Shao Y. (2017). Generation and Characterization of HIV-1 Transmitted and Founder Virus Consensus Sequence from Intravenous Drug Users in Xinjiang, China. AIDS Res. Hum. Retrovir..

[15] Wibmer C.K., Richardson S.I., Yolitz J., Cicala C., Arthos J., Moore P.L., Morris L. (2018). Common helical V1V2 conformations of HIV-1 Envelope expose the alpha4beta7 binding site on intact virions. Nat. Commun..

[16] Kwon Y.D., Pancera M., Acharya P., Georgiev I.S., Crooks E.T., Gorman J., Joyce M.G., Guttman M., Ma X., Narpala S. (2015). Crystal structure, conformational fixation and entry-related interactions of mature ligand-free HIV-1 Env. Nat. Struct. Mol. Biol..

[17] Lyumkis D., Julien J.P., de Val N., Cupo A., Potter C.S., Klasse P.J., Burton D.R., Sanders R.W., Moore J.P., Carragher B. (2013). Cryo-EM structure of a fully glycosylated soluble cleaved HIV-1 envelope trimer. Science.

[18] Julien J.P., Cupo A., Sok D., Stanfield R.L., Lyumkis D., Deller M.C., Klasse P.J., Burton D.R., Sanders R.W., Moore J.P. (2013). Crystal structure of a soluble cleaved HIV-1 envelope trimer. Science.

[19] McLellan J.S., Pancera M., Carrico C., Gorman J., Julien J.P., Khayat R., Louder R., Pejchal R., Sastry M., Dai K. (2011). Structure of HIV-1 gp120 V1/V2 domain with broadly neutralizing antibody PG9. Nature.

[20] Gorman J., Soto C., Yang M.M., Davenport T.M., Guttman M., Bailer R.T., Chambers M., Chuang G.Y., DeKosky B.J., Doria-Rose N.A. (2016). Structures of HIV-1 Env V1V2 with broadly neutralizing antibodies reveal commonalities that enable vaccine design. Nat. Struct. Mol. Biol..

[21] Cale E.M., Gorman J., Radakovich N.A., Crooks E.T., Osawa K., Tong T., Li J., Nagarajan R., Ozorowski G., Ambrozak D.R. (2017). Virus-like Particles Identify an HIV V1V2 Apex-Binding Neutralizing Antibody that Lacks a Protruding Loop. Immunity.

[22] Wang H., Gristick H.B., Scharf L., West A.P., Galimidi R.P., Seaman M.S., Freund N.T., Nussenzweig M.C., Bjorkman P.J. (2017). Asymmetric recognition of HIV-1 Envelope trimer by V1V2 loop-targeting antibodies. eLife.

[23] Pancera M., Zhou T., Druz A., Georgiev I.S., Soto C., Gorman J., Huang J., Acharya P., Chuang G.Y., Ofek G. (2014). Structure and immune recognition of trimeric pre-fusion HIV-1 Env. Nature.

[24] Wang H., Cohen A.A., Galimidi R.P., Gristick H.B., Jensen G.J., Bjorkman P.J. (2016). Cryo-EM structure of a CD4-bound open HIV-1 envelope trimer reveals structural rearrangements of the gp120 V1V2 loop. Proc. Natl. Acad. Sci. USA.

[25] Wang H., Barnes C.O., Yang Z., Nussenzweig M.C., Bjorkman P.J. (2018). Partially Open HIV-1 Envelope Structures Exhibit Conformational Changes Relevant for Coreceptor Binding and Fusion. Cell Host Microbe.

[26] Pancera M., Shahzad-Ul-Hussan S., Doria-Rose N.A., McLellan J.S., Bailer R.T., Dai K., Loesgen S., Louder M.K., Staupe R.P., Yang Y. (2013). Structural basis for diverse N-glycan recognition by HIV-1-neutralizing V1-V2-directed antibody PG16. Nat. Struct. Mol. Biol..

[27] Lee J.H., Andrabi R., Su C.Y., Yasmeen A., Julien J.P., Kong L., Wu N.C., McBride R., Sok D., Pauthner M. (2017). A Broadly Neutralizing Antibody Targets the Dynamic HIV Envelope Trimer Apex via a Long, Rigidified, and Anionic beta-Hairpin Structure. Immunity.

[28] Gorny M.K., Pan R., Williams C., Wang X.H., Volsky B., O’Neal T., Spurrier B., Sampson J.M., Li L., Seaman M.S. (2012). Functional and immunochemical cross-reactivity of V2-specific monoclonal antibodies from HIV-1-infected individuals. Virology.

[29] Liao H.X., Bonsignori M., Alam S.M., McLellan J.S., Tomaras G.D., Moody M.A., Kozink D.M., Hwang K.K., Chen X., Tsao C.Y. (2013). Vaccine induction of antibodies against a structurally heterogeneous site of immune pressure within HIV-1 envelope protein variable regions 1 and 2. Immunity.

[30] Wiehe K., Easterhoff D., Luo K., Nicely N.I., Bradley T., Jaeger F.H., Dennison S.M., Zhang R., Lloyd K.E., Stolarchuk C. (2014). Antibody light-chain-restricted recognition of the site of immune pressure in the RV144 HIV-1 vaccine trial is phylogenetically conserved. Immunity.

[31] Aiyegbo M.S., Shmelkov E., Dominguez L., Goger M., Battacharya S., deCamp A.C., Gilbert P.B., Berman P.W., Cardozo T. (2017). Peptide Targeted by Human Antibodies Associated with HIV Vaccine-Associated Protection Assumes a Dynamic alpha-Helical Structure. PLOS ONE.

[32] Pollara J., Bonsignori M., Moody M.A., Liu P., Alam S.M., Hwang K.K., Gurley T.C., Kozink D.M., Armand L.C., Marshall D.J. (2014). HIV-1 vaccine-induced C1 and V2 Env-specific antibodies synergize for increased antiviral activities. J. Virol..

[33] Peachman K.K., Karasavvas N., Chenine A.L., McLinden R., Rerks-Ngarm S., Jaranit K., Nitayaphan S., Pitisuttithum P., Tovanabutra S., Zolla-Pazner S. (2015). Identification of New Regions in HIV-1 gp120 Variable 2 and 3 Loops that Bind to alpha4beta7 Integrin Receptor. PLOS ONE.

[34] Robb M.L., Eller L.A., Kibuuka H., Rono K., Maganga L., Nitayaphan S., Kroon E., Sawe F.K., Sinei S., Sriplienchan S. (2016). Prospective Study of Acute HIV-1 Infection in Adults in East Africa and Thailand. N. Engl. J. Med..

[35] Eller M.A., Goonetilleke N., Tassaneetrithep B., Eller L.A., Costanzo M.C., Johnson S., Betts M.R., Krebs S.J., Slike B.M., Nitayaphan S. (2016). Expansion of Inefficient HIV-Specific CD8 T Cells during Acute Infection. J. Virol..

[36] Baden L.R., Walsh S.R., Seaman M.S., Cohen Y.Z., Johnson J.A., Licona J.H., Filter R.D., Kleinjan J.A., Gothing J.A., Jennings J. (2018). First-in-Human Randomized, Controlled Trial of Mosaic HIV-1 Immunogens Delivered via a Modified Vaccinia Ankara Vector. J. Infect. Dis..

[37] Rao M., Onkar S., Peachman K.K., White Y., Trinh H.V., Jobe O., Zhou Y., Dawson P., Eller M.A., Matyas G.R. (2018). Liposome-Encapsulated Human Immunodeficiency Virus-1 gp120 Induces Potent V1V2-Specific Antibodies in Humans. J. Infect. Dis..

[38] Vaccari M., Fourati S., Gordon S.N., Brown D.R., Bissa M., Schifanella L., Silva de Castro I., Doster M.N., Galli V., Omsland M. (2018). HIV vaccine candidate activation of hypoxia and the inflammasome in CD14(+) monocytes is associated with a decreased risk of SIVmac251 acquisition. Nat. Med..

[39] Wen Y., Trinh H.V., Linton C.E., Tani C., Norais N., Martinez-Guzman D., Ramesh P., Sun Y., Situ F., Karaca-Griffin S. (2018). Generation and characterization of a bivalent protein boost for future clinical trials: HIV-1 subtypes CR01_AE and B gp120 antigens with a potent adjuvant. PLOS ONE.

[40] Singh S., Ramirez-Salazar E.G., Doueiri R., Valentin A., Rosati M., Hu X., Keele B.F., Shen X., Tomaras G.D., Ferrari G. (2018). Control of Heterologous Simian Immunodeficiency Virus SIVsmE660 Infection by DNA and Protein Coimmunization Regimens Combined with Different Toll-Like-Receptor-4-Based Adjuvants in Macaques. J. Virol..

[41] Kijak G.H., Sanders-Buell E., Chenine A.L., Eller M.A., Goonetilleke N., Thomas R., Leviyang S., Harbolick E.A., Bose M., Pham P. (2017). Rare HIV-1 transmitted/founder lineages identified by deep viral sequencing contribute to rapid shifts in dominant quasispecies during acute and early infection. PLoS Pathog..

[42] Salazar-Gonzalez J.F., Bailes E., Pham K.T., Salazar M.G., Guffey M.B., Keele B.F., Derdeyn C.A., Farmer P., Hunter E., Allen S. (2008). Deciphering human immunodeficiency virus type 1 transmission and early envelope diversification by single-genome amplification and sequencing. J. Virol..

[43] Spurrier B., Sampson J., Gorny M.K., Zolla-Pazner S., Kong X.P. (2014). Functional implications of the binding mode of a human conformation-dependent V2 monoclonal antibody against HIV. J. Virol..

