# Correction: Trinh, H.V., et al. Humoral Response to the HIV-1 Envelope V2 Region in a Thai Early Acute Infection Cohort. *Cells* 2019, *8*, 365

**DOI:** 10.3390/cells8060554

**Published:** 2019-06-06

**Authors:** Hung V. Trinh, Neelakshi Gohain, Peter T. Pham, Christopher Hamlin, Hongshuo Song, Eric Sanders-Buell, Meera Bose, Leigh A. Eller, Swati Jain, Gherman Uritskiy, Venigalla B. Rao, Sodsai Tovanabutra, Nelson L. Michael, Merlin L. Robb, M. Gordon Joyce, Mangala Rao

**Affiliations:** 1 U.S. Military HIV Research Program, Walter Reed Army Institute of Research, Silver Spring, MD 20910, USA; htrinh@hivresearch.org (H.V.T.); ngohain@hivresearch.org (N.G.); ppham@hivresearch.org (P.T.P.); christopher.hamlin@nih.gov (C.H.); hsong@hivresearch.org (H.S.); esandersbuell@hivresearch.org (E.S.-B.); mbose@hivresearch.org (M.B.); leller@hivresearch.org (L.A.E.); Stovanabutra@hivresearch.org (S.T.); nmichael@hivresearch.org (N.L.M.); mrobb@hivresearch.org (M.L.R.); 2 Henry M. Jackson Foundation for the Advancement of Military Medicine, Inc., Bethesda, MD 20817, USA; 3 Department of Biology, The Catholic University of America, Washington, DC 20064, USA; 80jain@cua.edu (S.J.); guritsk1@jhu.edu (G.U.); rao@cua.edu (V.B.R.)

In the original version of our article [1], insufficient acknowledgement was given for initial analysis of RV217 participant 40007 HIV-1 viral sequences, and for identification of HIV-1 viral variants with His or Tyr at position 173 and a short deletion DSY in the V2 loop, likely due to host immune pressure that results in significant Env antigenic changes. We apologize for the original error. To correct this oversight, Swati Jain, Gherman Uritskiy, and Venigalla B. Rao have been added as authors, and the acknowledgements have been altered to appropriately recognize support and funding.

The corrected author list and author contributions are provided below:

Hung V. Trinh ^1,2,†^, Neelakshi Gohain ^1,2,†^, Peter T. Pham ^1,2^, Christopher Hamlin ^1,2,‡^, Hongshuo Song ^1,2^, Eric Sanders-Buell ^1,2^, Meera Bose ^1,2^, Leigh A. Eller ^1,2^, Swati Jain ^3^, Gherman Uritskiy ^3^, Venigalla B. Rao ^3^, Sodsai Tovanabutra ^1,2^, Nelson L. Michael ^1^, Merlin L. Robb ^1,2^, M. Gordon Joyce ^1,2,^* and Mangala Rao ^1,^*

^1^  U.S. Military HIV Research Program, Walter Reed Army Institute of Research, Silver Spring, MD 20910, USA; htrinh@hivresearch.org (H.V.T.); ngohain@hivresearch.org (N.G.); ppham@hivresearch.org (P.T.P.); christopher.hamlin@nih.gov (C.H.); hsong@hivresearch.org (H.S.); esandersbuell@hivresearch.org (E.S.-B.); mbose@hivresearch.org (M.B.); leller@hivresearch.org (L.A.E.); Stovanabutra@hivresearch.org (S.T.); nmichael@hivresearch.org (N.L.M.); mrobb@hivresearch.org (M.L.R.)
^2^  Henry M. Jackson Foundation for the Advancement of Military Medicine, Inc., Bethesda, MD 20817, USA
^3^  Department of Biology, The Catholic University of America, Washington, DC 20064, USA; 80jain@cua.edu (S.J.); guritsk1@jhu.edu (G.U.); rao@cua.edu (V.B.R.)
^*^  Correspondence: gjoyce@hivresearch.org (M.G.J.); mrao@hivresearch.org (M.R.); Tel.: +1-(301)-319-7528 (M.G.J.); +1-(301)-319-7699 (M.R.)
^†^  These authors contributed equally to this work.
^‡^  Current address: DAIDS, National Institute of Allergy and Infectious Diseases, Rockville, MD 20852, USA.
